# Co‐Designing a Primary Healthcare Intervention to Improve Diabetes Care in Mendoza, Argentina: A Qualitative Case Study

**DOI:** 10.1111/hex.70536

**Published:** 2025-12-23

**Authors:** Javier Roberti, Agustina Mazzoni, Marina Guglielmino, Cecilia Silva, Yanina Mazzaresi, Andrea Falaschi, Lisa R. Hirschhorn, John J. Parker, Ezequiel García‐Elorrio

**Affiliations:** ^1^ CIESP‐ CONICET Buenos Aires Argentina; ^2^ Institute of Clinical Effectiveness and Health Policy Buenos Aires Argentina; ^3^ Ministry of Health of Mendoza Mendoza Argentina; ^4^ Feinberg School of Medicine Northwestern University Chicago USA

**Keywords:** co‐design, diabetes care, implementation research, primary healthcare, stakeholder engagement

## Abstract

**Background:**

In low‐ and middle‐income countries, primary healthcare (PHC) faces significant challenges in delivering effective care for chronic conditions, exacerbated by fragmented systems, resource limitations, and inequitable access. In Argentina, despite national strategies for non‐communicable diseases, implementation varies due to decentralised governance, leading to gaps in care. Here, we describe the codesign process of a contextually relevant intervention to strengthen diabetes care in Mendoza's PHC system.

**Methods:**

Using a qualitative case study approach, we conducted co‐design workshops (11/2024 – 02/2025) involving patients, healthcare providers, and policymakers. Activities included user journey mapping, world café, and prioritisation exercises guided by the Implementation Research Logic Model and Normalisation Process Theory. We collected data from activities and were analysed using reflexive thematic analysis.

**Results:**

Participants (*n* = 38) identified systemic barriers, including insufficient resources, poor coordination, and patient access challenges. Emotional engagement and creative exercises, such as role‐playing, fostered collaboration and innovative problem‐solving. The codesigned package emphasised multidimensional strategies, stakeholder collaboration, and systemic improvements tailored to local needs. Some of the proposed strategies included community asset mapping and social prescribing, protected appointment slots, digitalising patient records.

**Conclusion:**

This study shows that a structured codesign process, informed by theory and previous research, can support the development of a context‐specific intervention to improve diabetes care in PHC. The workshops enabled the identification of feasible implementation strategies that reflected diverse stakeholder perspectives. While the effectiveness of the intervention will be tested in future phases, the co‐design approach was feasible and well‐received in this setting, offering insights for similar efforts in other contexts.

**Patient or Public Contribution:**

Patients, family caregivers, and primary healthcare users actively participated in the co‐design process, contributing to the identification of challenges in diabetes care and the development of context‐specific solutions. Their lived experiences informed the previous studies that were used for the codesign process, the design and prioritisation of strategies through structured activities. Participants also reviewed the proposed components and provided feedback that helped shape the final intervention package.

## 1 Introduction

Type 2 diabetes (T2D) is a public health challenge in Latin America, affecting nearly 10% of the population in Argentina and contributing significantly to morbidity and mortality [1, 2]. Despite widespread access to healthcare, many patients struggle with poor disease control, leading to preventable complications. Fragmentation within the healthcare system exacerbates these challenges, as wealthier individuals often seek private care while disparities in access persist based on income, education, rurality, and ethnicity [3, 4]. Effective management of T2D relies on continuous monitoring and strong patient‐provider interactions. By enhancing primary healthcare (PHC), timely interventions and adherence to treatment plans can be achieved, significantly reducing T2D complications and leading to better patient outcomes and satisfaction [1, 5]. Therefore, improving PHC is essential not only for optimising diabetes management but also for preventing complications associated with T2D.

In low‐ and middle‐income countries (LMICs), PHC should be the base of fair and efficient healthcare [6, 7, 8]. However, multiple challenges, such as fragmented and segmented systems, reduce its effectiveness [8, 9, 10, 11]. The limited availability of services provided by public and social security‐funded systems in these countries worsens the situation. However, the traditional model, which positions PHC as the primary contact for most health needs, is undermined by epidemiological shifts and increasing demands for highly effective care [12, 13]. In such a context, PHC often falls short, leading to hospitals treating patients with non‐communicable diseases (NCDs) that PHC providers could have managed. Integrating PHC necessitates substantial changes in financing, management, and delivery of health services [14, 15]. Efforts and interventions to enhance PHC management of NCDs have seen notable progress through integrated care models and multidisciplinary teams; however, the management of diabetes often lags behind [16, 17, 18, 19, 20].

In Argentina, the implementation of national strategies for NCDs has been uneven due to the decentralised nature of the healthcare system. While the national framework provides strategic direction, its execution depends largely on provincial political will, leading to variability in care delivery. Mendoza exemplifies these challenges. Despite the availability of PHC services, many patients with NCDs continue to seek care at hospitals, often due to perceived gaps in primary‐level services, including long wait times and limited diagnostic and treatment capacity. In 2022, Mendoza's Ministry of Health collaborated with the Quality Evidence for Health System Transformation (QuEST) network to develop and evaluate an intervention aimed at improving diabetes care at the primary level. As part of the QuEST network, our team has documented inequities in care across multiple Latin American countries, identified and addressed gaps in patient‐reported experience measurement through innovative assessment methods, and worked to align researchers and policymakers across the region [21, 22]. The design of such interventions is a critical first step in the development‐evaluation‐implementation continuum outlined by the Medical Research Council framework [23]. Given the complexity of health system redesign, we adopted a co‐design approach, engaging patients, providers, and policymakers to ensure that proposed solutions are contextually relevant, feasible, and aligned with local needs. This participatory process strengthens the intervention's effectiveness and promotes equity by integrating diverse stakeholder perspectives from the outset.

Community engagement involves collaborating with community stakeholders to identify and implement solutions to health challenges [24, 25]. This inclusive process improves the acceptability, feasibility, and local relevance of interventions, building trust and credibility between communities and researchers. Co‐designing health initiatives with community members ensures they align with local priorities, leading to more effective and sustainable outcomes [24, 25, 26]. Co‐production was the approach we used to develop an intervention to improve PHC. Co‐production emerged as a response to the challenges of translating research into practice, emphasising knowledge generation within its real‐world context. By actively involving staff and patients, co‐production ensures that health services are shaped by those who use and deliver them, enhances the cultural appropriateness of health interventions and creates a sense of ownership among stakeholders [27]. Co‐design is a distinct activity within co‐production, aimed at building capacity and enhancing public service delivery [28]. Based on user‐centred and human‐centred design, its application in healthcare gained importance with the emergence of Experience‐Based Co‐Design (EBCD) in the 2000s [28]. Co‐design techniques acknowledge the emotional dimensions of experience, encourage meaningful connections between groups, strengthen engagement, and create a shared space for discussing common interests [29].

This paper documents the co‐design process of a complex intervention to strengthen diabetes care in Mendoza's PHC system, ensuring its feasibility, relevance, and alignment with local needs. Detailing the strategies used to engage key stakeholders provides a practical framework for similar initiatives. Additionally, it highlights the challenges and facilitators encountered during the process, offering valuable insights for improving the implementation of health interventions, particularly in resource‐constrained settings. By sharing this experience, we contribute to the growing body of evidence on co‐design methodologies in healthcare and their potential to enhance the effectiveness, sustainability, and equity of primary care interventions for chronic disease management.

## 2 Methods

### 2.1 Design

This study uses a qualitative case study approach to describe the co‐design process of a complex intervention to improve T2D care, building on what we identified as gaps in PHC in Mendoza. Co‐design activities were conducted through in‐person workshops in Mendoza from November 2024 to February 2025. We adopted Stake's instrumental case study approach, utilising the co‐design process as a means of gaining insights into how co‐design methodologies can be applied to develop interventions in comparable settings [30]. These workshops brought together patients, healthcare providers, and policymakers to collaboratively identify challenges, propose solutions, and refine strategies for diabetes management. The resulting intervention will be tested in a pilot and then a randomised trial to assess its feasibility and effectiveness.

### 2.2 Setting

Argentina's health system has three subsectors: public, social security, and private. The Ministry of Health funds the public sector, serving uninsured people from lower socioeconomic groups (36%). The social security sector covers 60% of the population and requires all employers and employees to pay into a trust fund. The private sector provides services to individuals of high socioeconomic status who pay for health insurance packages. In 2009, the National Strategy for the Prevention and Control of NCDs was created to reduce NCD risk factors and enhance care quality and access. Mendoza, a province with 2 million people, faces challenges in treating NCDs such as T2D at the PHC level. Hospitals still treat patients with NCDs that PHC can manage. Gaps include long waiting times, scheduling problems, and a lack of trust in some providers. Additionally, PHC providers often lack confidence in diagnosing and treating NCDs and have insufficient medication and diagnostic tests.

### 2.3 Participants

Participants in workshops included individuals living with T2D and managing the disease in the public PHC system, healthcare professionals working in public PHC and providing care for people with diabetes, and decision‐makers from the Ministry of Health of Mendoza. Healthcare professionals were approached by researchers from the Ministry of Health, who had been involved in the project since 2021. Then, these professionals recruited patients with T2D at their centres in Mendoza and semi‐rural areas near the city of Mendoza. All participants were provided with verbal, written, or electronic information about the study and workshops, and they signed an informed consent form. To acknowledge the contributions of patients, compensation was provided in the form of a USD40 supermarket gift card, along with transportation coverage to facilitate their participation. Transportation costs were also covered for healthcare professionals and decision‐makers. Professionals were issued participation certificates if requested.

### 2.4 Material Informing the Co‐Design Process and Framework

The co‐design process was informed by prior research conducted in Mendoza, the Implementation Research Logic Model (IRLM), and Normalisation Process Theory (NPT) to map out relationships between determinants, strategies, mechanisms, and outcomes. Table 1 provides an overview of the key research components that led to this co‐design process, including studies on healthcare utilisation, patient experiences, system challenges, and stakeholder perspectives. Findings from these research components were strategically incorporated into the workshops to guide discussions.

**Table 1:** Previous studies the group conducted in Mendoza which led to this codesign process.

Component	Objective
Evaluation of patterns of care	Assessed health system utilisation and disease control levels.
Focus groups with patients	Evaluated burden of treatment for patients with chronic conditions.
In‐depth interviews with stakeholders	Assessed program activities, services, and system challenges.
People's Voice Survey	Measured user interaction and perception of the health system.
Electronic cohort of diabetic patients	Evaluated diabetes management in the health system.
Knowledge test for healthcare providers	Identified weaknesses in provider skills for chronic disease care.
Consensus process for recommendations	Developed evidence‐based recommendations for primary care improvement.

The IRLM served as a conceptual framework to structure the co‐design process by mapping the relationships between implementation determinants, strategies, mechanisms of action, and outcomes [31]. This model emphasises three key principles: (a) intervention strategies must be tailored to context‐specific barriers and facilitators, (b) selected strategies function through distinct mechanisms to drive change, and (c) implementation outcomes, shaped by these mechanisms, ultimately influence clinical results. Within this framework, implementation determinants represent factors that either support or obstruct the adoption of interventions [31]. Implementation strategies (ISs) are modifications and system‐level supports designed to facilitate the integration of evidence‐based practices into routine care. Mechanisms of action describe the processes through which these strategies achieve their intended effects [31]. Finally, implementation outcomes assess the effectiveness and sustainability of the intervention and its impact on service delivery and clinical care. Using the IRLM in this study's planning phase, we identified context‐specific challenges, feasible ISs, and their underlying mechanisms [31]. NPT guided the expansion of ISs into specific, actionable activities by helping us anticipate the types of work required for successful integration. We considered coherence by ensuring all stakeholders shared a clear understanding of the intervention's purpose and value [32, 33]. We promoted cognitive participation through collaborative design sessions that encouraged active engagement and ownership. We addressed collective action by identifying the practical tasks and roles needed for implementation. Finally, we planned for reflexive monitoring by establishing mechanisms for evaluation and adaptation based on feedback. This theoretical lens helped structure the process, even if not explicitly referenced in each step [32, 33].

### 2.5 Workshop Organisation

We considered several key factors for the workshops to ensure a conducive and interactive environment. The workshops were held at venues provided by the Ministry of Health, where breakfast and lunch were offered to all attendees to support a welcoming and collaborative environment. We selected a large room that allowed for easy movement of tables and chairs for participants and had enough space for a table for a computer and a projector. The workshops were facilitated by five people (two researchers, a psychologist, an acting teacher, and a social worker). Participants were divided into heterogeneous groups, ensuring diverse perspectives were incorporated into the discussions. After the group activities, facilitators summarised key points from each group and presented them for broader discussion. Below, we provide a detailed account of the workshops, including their structure and activities.

### 2.6 Codesign Workshop Process

#### 2.6.1 Workshop 1

**Figure 1:**
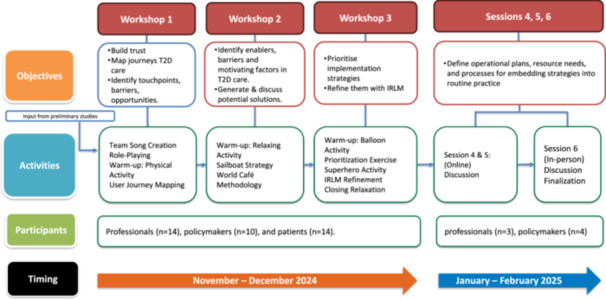
Codesign process timeline, objectives and activities.

The initial session lasted 2 h and was centred on exchanging experiences and outlining the journey of patients, professionals, and decision‐makers in T2D care. The workshop started with activities that promoted trust, mutual respect, and group cohesion through interactive engagement. Participants were introduced to the research project, and the main barriers in T2D as informed by previous research (Table 1). The following activity, *role‐playing*, aimed to encourage participants to reflect on personal experiences related to T2D and foster empathy among them. Facilitators provided each team with a scenario and prompts to highlight real‐life issues faced by different stakeholders in T2D care. Participants in heterogeneous groups were invited to write a script that represented the interactions they were often part of. Then, they performed those interactions. The following activity was a user journey mapping exercise. Participants were divided into three groups: patients, healthcare professionals, and policymakers. Each group was tasked with creating a user journey diagram that captured the experiences of diabetes care within the PHC system. The diagram highlighted key touchpoints, emotions, barriers or pain points, and opportunities for improvement across phases of care in posters to represent the journeys visually. After the activity, each group organised their findings to compare perspectives, highlighting shared challenges and unique insights across stakeholder roles (Figure 1). The research team analysed notes and outputs to use them as inputs for the subsequent workshop.

#### 2.6.2 Workshop 2

**Figure 2:**
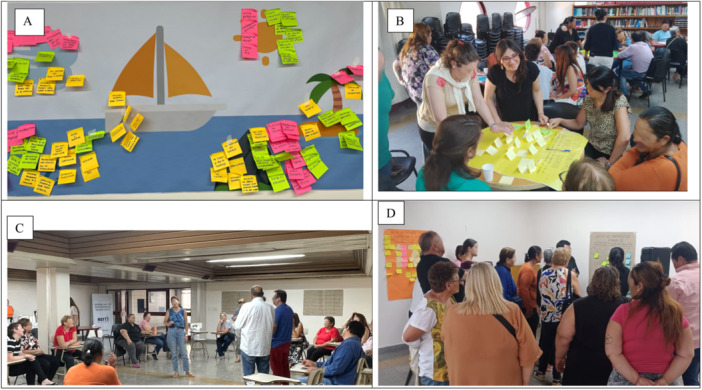
(A) Sailboat strategy. (B) World café activity. (C) Warm‐up activity. (D) Group of patients prioritising interventions. *Note:* Participants´ faces have been blurred to preserve privacy.

During the second workshop, participants engaged in activities to identify the key facilitators and barriers to improving diabetes care. These activities built on the broad themes outlined in the first session. The workshop began with a warm‐up activity to create a welcoming and participatory environment. This was followed by the Sailboat Strategy exercise, which used a metaphorical sailboat to help participants explore, identify, and categorise factors influencing care for T2D. The boat represented the current diabetes care system, navigating toward optimal care. Patients, healthcare professionals, and decision‐makers classified elements as facilitators (wind), structural barriers (anchor), intermittent barriers (rocks), motivating factors (sun), and the ideal care model, the full adoption of the evidence‐based clinical guideline (island) (Figure 2). The second activity applied the World Café methodology to deepen the discussion around the problems and opportunities identified in the sailboat exercise. Each thematic table corresponded to one of four areas: healthcare delivery, patient education, adherence to care, and follow‐up. Participants were arranged into diverse groups—each including patients, professionals, and decision‐makers—and rotated through all four tables. This process ensured that each area received input from all stakeholder perspectives. Trained facilitators guided the discussions at each table. At the end of the session, facilitators synthesised the results across tables, summarising key problems and proposed solutions to these problems, as presented in the Results section.

#### 2.6.3 Workshop 3

On the third day, participants focused on prioritising and refining the solutions proposed at the end of the previous workshop and developing a practical implementation plan using elements from the IRLM [31]. As a warm‐up activity, participants formed a circle and engaged in a dynamic involving balloons, each representing important aspects of T2D. The goal was to work collectively to keep the balloons in the air, symbolising the need to prevent disinterest or abandonment of care. Then, participants were divided into three groups: patients, healthcare providers, and policymakers. They reviewed posters from the last workshop and had to identify and prioritise the proposed solutions, transforming them into actionable ISs. Workshop facilitators introduced two prioritisation criteria: impact and feasibility. The three groups rotated through stations, discussed, and ranked all strategies using different colours. This activity resulted in a ranked list of ISs that would form the core components of the intervention package.

The following activity, “superhero,” aimed to encourage reflection on capacities and needs through imaginative play. Facilitators guided the creation of a superhero character capable of solving all diabetes‐related problems with specific superpowers, such as the superhuman ability to obtain appointments. The superhero was then demonstrated in a particular situation, fostering collaborative problem‐solving. Then, with the results from the prioritisation exercise, participants were introduced to a simplified version of the IRLM for the following exercise. Each group chose two prioritised ISs and, over 60 min, collaboratively identified barriers and facilitators to a specific strategy, proposed how to address them, and defined mechanisms of action to achieve outcomes. Facilitators rotated among groups to ensure progress and clarify questions. Then, each group presented its refined IRLM for the chosen strategies to the plenary. Finally, a relaxation activity was the closing exercise for the codesign workshops.

#### 2.6.4 Sessions 4–6

Sessions 4–6 were conducted with a smaller group of participants; healthcare professionals and decision‐makers who had taken part in previous workshops. Patients were not included in these sessions. The focus had shifted to refining the co‐designed ISs by addressing logistical, organisational, and operational aspects that required technical expertise and decision‐making authority. These sessions aimed to transform the set of proposed strategies into a cohesive and feasible implementation package. Participants collaboratively prioritised strategies, and discussions focused on resource requirements, service coordination, and alignment with the existing health system. The IRLM was employed to map each strategy's core components—such as determinants, mechanisms, and expected outcomes, ensuring internal coherence and clarity. In parallel, NPT guided reflection on the processes needed to embed these strategies into routine practice, helping identify potential challenges to collective action and long‐term sustainability. An implementation planning document was developed and iteratively refined over 3 weeks, incorporating participants' input on each selected strategy, including key actions and required material and human resources.

### 2.7 Data Collection and Analysis

Facilitators collected data in written form through detailed notes and summary reports during and after each workshop, documenting key discussions and emerging insights related to intervention development. In addition, completed workshop materials, such as templates, posters, and photographs of activities, were included as data sources. A pragmatic, reflexive thematic analysis approach was applied to analyse these data [34]. First, descriptive codes were generated from the notes and visual materials, followed by the identification of more interpretive, latent codes through iterative team discussions. Subsequently, we conducted a theory‐informed analysis of the co‐design outputs, drawing on relevant constructs from NPT to guide interpretation. Themes were refined through collaborative research meetings, resulting in a set of meaning‐based themes that informed the final intervention package. Concerning reflexivity and positionality, some research team members had previously worked within the health system in Mendoza, which facilitated access and trust but also required attention to power dynamics during the process. While the research team designed the workshop structure and selected participatory methods, the substantive content, including solution generation and prioritisation, was entirely participant‐driven. The facilitators' role was to enable discussion and ensure equitable participation, not to guide or influence the direction of proposed strategies. Throughout the workshops, we prioritised creating a safe space where all voices, especially those of patients, were valued equally and where participants, not facilitators or research team members, determined the priorities and solutions. We regularly reflected on our assumptions about PHC and care, recognising that our training or experience might differ from lived experience perspectives. This reflexive stance informed our facilitation approach and analytical process, helping us be attentive to whose voices might be privileged.

### 2.8 Ethics

Ethical approval for the redesign project was obtained from the Provincial Council for the Ethical Evaluation of Health Research (CoPEIS), Department of Research, Science, and Technology of the Ministry of Health and Sports of Mendoza, Resolution No. 149/2024. The workshops followed a co‐design methodology where all stakeholders equally participated in developing a new intervention. Written consent to participate in the workshops and to appear in photographs was obtained from all participants, and comprehensive information on the research process was provided.

## 3 Results

### 3.1 Participants Characteristics

The co‐design workshops included a diverse group of participants, comprising healthcare professionals (*n* = 14), decision‐makers (*n* = 10), and patients (*n* = 14). Among the healthcare professionals were family physicians, diabetologists, nurses, nutritionists, and community health agents. Decision‐makers included directors and coordinators of primary care centres, regional health authorities, and Ministry secretaries.

#### 3.1.1 Workshop 1. Stakeholders' Journey

The first workshop marked the beginning of a collective reflection on the diabetes care experience from multiple perspectives. Through role‐playing and guided discussions, patients, healthcare professionals, and decision‐makers shared their respective “journeys” across the diabetes care continuum. This activity revealed key pain points, emotional responses, and systemic barriers encountered by each group, as well as preliminary ideas for improvement. Below, we present a synthesis of these journeys, illustrating how each stakeholder experiences and navigates the challenges of diabetes management within the current PHC system.

**Professionals' journey.** Healthcare professionals outlined a journey that included screening, diagnosis, follow‐up, and adherence support. They identified significant challenges, including the unavailability of HbA1C tests in all centres, high rates of appointment absenteeism, and the spread of misinformation through social media, which complicated patient education. These challenges often elicited frustration and anger over systemic barriers such as medication shortages, though moments of satisfaction were also noted. To address these issues, professionals proposed protected appointments for diabetes care, enhanced coordination with higher‐level care, and increased healthcare agents to improve service delivery and patient outcomes (Table 2).

**Table 2:** Main characteristics of the challenges and solutions in T2D care from the perspectives of healthcare professionals, decision‐makers, and patients.

Group	Journey stages	Challenges	Emotions experienced	Proposed solutions
Healthcare professionals	a. Screening, b. Diagnosis, c. Follow‐up, d. Adherence support	Unavailability of HbA1C tests High rates of appointment absenteeism Spread of misinformation through social media	Frustration, anger, moments of satisfaction	Protected appointments for diabetes care Enhanced coordination with higher‐level care Increased healthcare agents
Decision‐makers	a. Information gathering b. Planning c. Implementing policies d. Ensuring sustainability of T2D care	Absence of clear process and outcome indicators Insufficient supplies for treatment Poor coordination with secondary/tertiary care	Frustration, anguish, helplessness	Centralised management of diagnostic practices Improved logistics for resource distribution Stronger coordination mechanisms with higher‐level care
Patients	a. Recognising need for care, b. Diagnosis, c. Routine monitoring, d. Treatment adherence	Difficulties in securing timely appointments Forgetting to take medication Economic barriers to accessing care	Denial, fear of mortality, worry	Enhanced care quality Special diabetes cards for service access Efficient appointment scheduling systems Increased economic support

**Decision‐makers' journey.** Decision‐makers described their role in gathering information on T2D in the province, planning resource allocation, implementing policies, and ensuring the sustainability of T2D care. In this journey, they faced structural challenges, such as the absence of transparent processes and outcome indicators, difficulties in managing treatment due to insufficient supplies, and poor coordination with secondary and tertiary care levels. Decision‐makers reported anguish and helplessness due to structural inefficiencies and logistical constraints. To overcome these barriers, decision‐makers suggested centralised management of diagnostic practices, improved logistics for resource distribution, and stronger coordination mechanisms with higher‐level care to ensure continuity and quality of care (Table 2).

**Patients' journey.** Patients shared their journey, which included recognising the need for care because of signs or symptoms that may indicate T2D, undergoing tests to confirm a diagnosis, routine monitoring of T2D, and maintaining treatment adherence. Along their journey, patients faced challenges like securing appointments, remembering to take medication, and economic barriers to accessing care, such as transportation costs to hospitals or medication not covered by insurance. Patients explained they experienced a spectrum of emotions, from denial and fear upon diagnosis to frustration with appointment delays and financial burdens. Patients identified opportunities for improvement, including enhanced care quality, the introduction of special diabetes cards to facilitate access to services, more efficient appointment scheduling systems, and increased economic support to alleviate financial burdens (Table 2).

#### 3.1.2 Workshop 2. Barriers to Better T2D Care and Potential Strategies

In Workshop 2, building on the barriers identified through the journey mapping exercise in Workshop 1, participants expanded and refined their analysis by considering additional challenges from the perspectives of various subgroups. They further developed, categorised, and contextualised these barriers to link them with potential ISs. Table 3 shows the output of this workshop with categorised barriers to T2D care and proposed strategies to address them.

**Table 3:** Categorised barriers to T2D care and proposed strategies to address them.

Dimension	Barriers	Proposed strategies
Access to the healthcare system	Lack of appointments at secondary and tertiary care levels	Network coordination and collaboration across levels
	Lack of specialist appointments	Protected appointment slots managed from PHC centres
	Inequitable appointment allocation	Optimise scheduling based on population served
	Malfunctioning appointment hotline	Continuous training for operators
	Inadequate supply of medication	Improve patient registration and digitalisation
	Lack of connectivity	Implement telemedicine
Community support	Lack of safe walking spaces	Design secure circuits with municipalities
	Insufficient physical activity	Promote exercise programs in community centres
	Lack of family support	Establish support workshops and self‐help groups
	Medication adherence issues	Distribute weekly pill organisers
Patient care	Brief consultation time	Increase allocated consultation time
	Appointment absenteeism	Implement WhatsApp reminders
	Lack of patient‐centred care	Training workshops on person‐centred communication
System sustainability	Staff burnout	Conduct job satisfaction surveys
	Lack of statistical data within programs	Digitalise primary care centres (CAPS)
	Shortage of specialised human resources	Promote family medicine residencies

### 3.2 Access to the Healthcare System

Barriers to accessing the healthcare system included limited appointment availability at secondary and tertiary care levels and specific laboratory tests, inadequate technological connectivity in some geographical areas, which affected some PHC centres and their capacity to use electronic records and appointment systems, and deficiencies in medication supply. Proposed strategies included healthcare network coordination across levels, protected appointments with specialists managed from PHC centres, digitalisation of patient records, and the implementation of telemedicine supported by equipment acquisition and local technical assistance. Problems with the appointment hotline could be partially solved with continuous operator training and regular team meetings.

### 3.3 Patient Care

Patients perceived that consultation times were too brief regarding care, limiting their ability to discuss concerns in depth. Healthcare providers, on the other hand, identified appointment absenteeism as a key challenge that affected the continuity of care. Finally, decision‐makers highlighted the lack of a truly patient‐centred approach as the main barrier in this area, noting that healthcare services often fail to adequately consider patients' needs, preferences, and experiences when designing and delivering care. Proposed solutions included extending consultation durations, implementing WhatsApp reminders to reduce absenteeism, and providing training workshops on person‐centred communication. These initiatives were reinforced by continuous training and engagement with local opinion leaders.

### 3.4 Community Support

Participants identified several barriers, including the lack of safe pedestrian areas, limited community opportunities for physical activity, and insufficient familial support for elderly patients. Decision‐makers also noted challenges in collaborating with community stakeholders, particularly municipalities. They explained that when a different political party governs a city than the provincial government, cooperation becomes more difficult. To address these issues, participants recommended the development of secure walking circuits in partnership with municipalities and implementing community‐based physical activity programs in coordination with local entities such as clubs, churches, pensioner associations, and schools. Participants indicated that diverse institutions and alignment of the mayor's political affiliation with the provincial government in specific neighbourhoods served as a substantial enabler for any initiatives. Other suggested strategies included workshops for patients' families, as they were crucial for promoting adherence to treatment and overall well‐being, self‐help groups, and the distribution of weekly pill organisers. Local needs assessments, mass media dissemination, and patient feedback mechanisms should support these initiatives.

### 3.5 System Sustainability

Participants highlighted several challenges, including staff burnout, insufficient statistical data to inform decision‐making, and a shortage of specialised healthcare professionals, all threatening the system's long‐term viability. Suggested interventions included job satisfaction surveys, leadership training, digitalisation of primary care centres, and promoting family medicine residencies via academic partnerships and capitated payment schemes.

### 3.6 Workshop 3 and Subsequent Sessions: ISs to Improve T2D Management in PHC

In workshop 3, participants worked to prioritise the list of barriers and potential strategies to address those barriers (Table 3). Once a list of strategies was obtained, participants used the IRLM to develop the ideas further. These strategies included community assets mapping and social prescribing, optimising appointment systems, promoting self‐care, training healthcare professionals, improving patient registration, and leveraging digital tools for communication. During the refinement and operationalisation of the strategies, we used NPT constructs of coherence, cognitive participation, collective action, and reflexive monitoring to elucidate the underlying processes that will facilitate the normalisation of the IS into routine practice. Table 4 details this breakdown, illustrating how each strategy is operationalised to facilitate its eventual normalisation into routine practice.

**Table 4:** Final list of implementation strategies and activities.

Strategy	Objective	Normalisation process theory construct	Activities
Community assets mapping and social prescribing	Establish a community support network to integrate local resources into patient care plans.	Coherence	Raise awareness among directors and professionals through targeted briefings. Create and disseminate a community asset map with visible resource guides.
		Collective Action	Conduct interviews with stakeholders to map community initiatives. Establish monthly community management meetings with a facilitator.
Protected and deferred appointments	Establish dedicated appointment system for patients with T2D, ensuring timely access to specialists and tests.	Collective Action	Implement deferred appointment systems (manual or digital). Allocate protected slots for testing, follow‐ups, and annual dental check‐ups.
		Reflexive Monitoring	Address no‐show risks by deploying reminder systems and reviewing appointment logs. Monitor adherence to appointment scheduling using centre records.
Self‐care workshops for patients and families	Empower patients and families with knowledge and skills for effective disease management.	Cognitive Participation	Organise workshops and conduct monthly sessions across PHCs. Implement family‐focused workshops with psychological support.
		Coherence	Develop clear, core content for interactive learning that communicates the intervention's objectives.
		Reflexive Monitoring	Collaborate with ministry to refine themes based on feedback. Administer surveys at workshop exit to capture real‐time feedback
Professional training	Enhance healthcare providers' knowledge and skills in diabetes care to improve patient outcomes.	Coherence	Deliver in‐person training courses by certified professionals. Provide virtual training sessions aligned with national guidelines.
		Cognitive Participation	Develop tailored training sessions for different provider groups, ensuring content relevance and trainer neutrality.
		Collective Action	Provide ongoing support with external mentorship and schedule follow‐up mentor visits to reinforce training uptake.
Patient registration	Standardise and improve patient registration for better data management and care.	Collective Action	Assign data entry responsibilities to designated staff and enable self‐registration via QR codes/Google Forms. Facilitate patient–physician identification via WhatsApp (as per checklist guidance).
		Reflexive Monitoring	Use reports and manual/digital records to track patient registration; update records every 15 days as per centre protocol.
Use of WhatsApp for communication and engagement	Improve patient engagement, appointment adherence, and self‐care via WhatsApp.	Collective Action	Provide centres with a dedicated mobile phone with WhatsApp and a prepaid SIM.
		Reflexive Monitoring	Send appointment reminders and health education messages regularly. Create patient groups for information sharing and establish a community WhatsApp group for ongoing engagement.

## 4 Discussion

This study demonstrated the feasibility and value of a co‐design approach to developing interventions for improving diabetes care in primary care settings. Through an iterative process involving patients, healthcare providers, and decision‐makers and building on previous work, we identified key priorities. These included the need for personalised care pathways, digital tools to support self‐management, and strategies to address structural barriers to implementation. The workshops highlighted challenges: disparities in access to care, the role of family and peer support, and the need to ensure equitable representation of all voices in the design process. Notably, creative exercises played an important role in promoting engagement and discussions. These findings align with evidence on co‐design in diabetes care, reinforcing its potential to produce contextually relevant, patient‐centred interventions.

A review identified four main reasons for adopting co‐production approaches in healthcare: bringing people together, valuing all knowledge, producing more relevant research, and improving health outcomes [35]. These were facilitated by mechanisms such as meeting stakeholders' needs and fostering trust in the process. However, there was limited evidence that co‐production directly improved health outcomes due to a lack of robust intervention evaluations. Despite this, co‐production appears to play a crucial role in mobilising knowledge for health condition management [35]. A bibliometric analysis of co‐production in healthcare highlights its rapid growth, but despite this expansion, the field remains highly fragmented [36].

An essential strength of the co‐design approach is its ability to adapt interventions to the specific needs of diverse populations. Agarwal et al. [37] demonstrated how co‐design could drive equity‐focused solutions by leveraging diverse stakeholder perspectives with interventions aimed at increasing the uptake of diabetes technology among underserved populations. In Denmark, a culturally tailored education and support intervention significantly improved clinical outcomes among immigrant patients, demonstrating the importance of addressing language and cultural aspects in intervention design [38]. Similarly, a co‐design study developing SMS‐based health interventions for Hispanic adolescents showed that messages must align with the psychological needs and preferences of the target population to enhance engagement and effectiveness [39].

Several studies have reported the use of co‐design approaches to develop interventions aimed at improving diabetes care [39, 40, 41, 42, 43, 44, 45]. Common characteristics of cocreation processes in T2D care interventions include collaboration between diverse stakeholders, iterative feedback loops to refine ideas, and cultural and individual tailoring of solutions [41, 42, 44, 46]. These processes often involve user‐centred design to ensure usability and relevance, with a focus on technology integration for digital solutions [41, 42, 44, 46]. Tay et al. used a co‐design process to develop a digital dietary intervention for adults at risk of T2D, prioritising stakeholder engagement and integrating persuasive principles [42]. Similarly, Shetty et al. and Alvarez‐Perez et al. applied co‐design to develop mobile health applications for diabetes, incorporating personalised feedback and exercise guidance to improve user engagement [46, 47]. Some studies report improved clinical outcomes because of codesigned strategies and interventions [42, 47]. This reflects a broader trend in co‐design research, where process evaluation often takes precedence over outcome measurement.

Strategies often naturally emphasise certain implementation mechanisms over others depending on their specific aims and the context in which they are applied. In co‐design contexts, NPT can be used both to structure stakeholder discussions and to analyse how proposed strategies are likely to be integrated into routine practice. Its constructs help stakeholders articulate what the intervention is and why it matters, but also how it will be adopted, enacted, and sustained in real‐world settings. When embedded in co‐design workshops, NPT can make visible the work and relational dynamics required for implementation, supporting the creation of strategies that are technically sound and socially workable [48, 49]. This theory‐informed approach contributes to a more realistic, context‐sensitive planning, enhancing the chances of successful normalisation in complex health systems [48, 49]. Using NPT as an analytical lens allowed for the identification of potential gaps in the design and implementation of each strategy. If a strategy lacks activities related to, for example, coherence, this may indicate a need for adjustment to ensure that stakeholders can fully understand the strategy. Although it is not essential for every IS to include activities across all four implementation mechanisms, the overall IS package collectively addresses all aspects of normalisation.

Several key lessons emerged from our co‐design process. First, engaging stakeholders unfamiliar with this approach posed challenges. Some participants, particularly patients, initially expected a more traditional educational format rather than an interactive, collaborative process in which their voices carried equal weight. This lack of prior experience with co‐design affected their participation, showing the need for clearer facilitation strategies. Second, structural and systemic barriers remained a concern throughout the process. These factors could undermine the feasibility of any co‐designed intervention. Another challenge was the presence of potential biases within group dynamics, particularly the dominance of certain voices despite facilitation efforts to promote balanced participation. The iterative planning of workshop sessions ensured that all participants felt prepared and safe to share their perspectives and allowed the research team to refine strategies. Flexibility and inclusivity were critical in adapting to emerging needs and ensuring diverse perspectives. Finally, an unexpected insight emerged from the use of creative exercises, such as role‐playing and the superhero activity. Initially met with scepticism, these methods significantly enhanced engagement and encouraged participants to think beyond conventional problem‐solving approaches.

Despite challenges, our findings from this co‐design process may have relevance for similar PHC contexts across other LMICs with comparable problems. We distinguish between two types of transferability: the methodological approach and the specific ISs developed. This paper provides detailed documentation of our co‐design process to support researchers and practitioners seeking to facilitate similar processes in their own contexts. The value of this methodological contribution stands independent of whether the co‐designed strategies prove effective. We anticipate that challenges and necessary refinements will emerge, as is typical with complex interventions. However, the systematic application of theory‐informed co‐design, including the use of creative exercises, heterogeneous stakeholder groups, and structured prioritisation activities, offers a replicable framework for intervention development. However, important contextual differences must be considered, including political structures, health financing mechanisms, and cultural factors, among others. Many countries in the region share similar system characteristics, including fragmentation, decentralised governance structures, and resource constraints [50, 51]. The strategies co‐designed in this study, particularly the emphasis on digitalisation, protected appointment slots, and community assets mapping along with social prescribing, may be adaptable to these contexts. Our findings also highlight the role of political alignment and inter‐sectoral collaboration in implementation success. This suggests that attention to local governance dynamics is essential. Future research should explore how the co‐design methodology and ISs perform when adapted to different settings.

While this study employed a comprehensive and participatory co‐design process grounded in theory and prior research, several methodological limitations should be acknowledged. The recruitment of patients through healthcare professionals may have introduced selection bias, favouring individuals with more frequent engagement or positive relationships with the health system. Additionally, participating patients were already connected to the PHC system, potentially excluding the perspectives of more marginalised or disengaged individuals. To mitigate this, we incorporated findings from the People's Voice Survey, a province‐wide telephone survey exploring how users and non‐users relate to the health system. This helped ensure that the co‐design process was informed by a broader range of experiences. Additionally, during the trial, community engagement efforts will be conducted to ensure that disadvantaged populations are reached. Furthermore, patients did not participate in the final refinement sessions, which focused on operational planning with healthcare professionals and decision‐makers. Although appropriate for this stage, this may have excluded valuable user perspectives on feasibility and acceptability. Nevertheless, the workshop content was informed by diverse prior research conducted in Mendoza. This included studies on healthcare use, patient experiences, system barriers, and a Delphi consensus process, and involved a broad range of stakeholders. This strengthened the rigour and relevance of the resulting ISs. These strategies will be further evaluated in a forthcoming feasibility and effectiveness study.

This study shows the potential of the co‐design method to develop person‐centred interventions in primary care. By actively involving diverse stakeholders, the process facilitated the identification of context‐specific challenges and opportunities. It shaped ISs that could be inserted into the existing model but addressed gaps in service delivery. Despite challenges such as structural constraints and a lack of experience in this method, this iterative and inclusive approach ensured that the proposed interventions reflected real needs. Building on this work, we will first conduct a pilot study to assess the feasibility and refine the intervention package. This will be followed by a cluster‐randomised controlled trial to evaluate its effectiveness in primary care centres in Mendoza. Future efforts should scale and adapt the method and interventions to different settings and conditions, enhancing participation and impact.

## Author Contributions

**Javier Roberti:** conceptualisation, methodology, formal analysis, investigation, project administration, supervision, writing – original draft, writing – review and editing. **Agustina Mazzoni:** methodology, investigation, project administration, writing – review and editing. **Marina Guglielmino:** methodology, investigation, writing – review and editing. **Cecilia Silva:** conceptualisation, methodology, formal analysis, investigation, writing – review and editing. **Yanina Mazzaresi:** investigation, project administration, writing – review and editing. **Andrea Falaschi:** investigation, project administration, writing – review and editing. **Lisa R. Hirschhorn:** methodology, funding acquisition, writing – review and editing. **John J. Parker:** methodology, funding acquisition, writing – review and editing. **Ezequiel García‐Elorrio:** conceptualisation, methodology, investigation, resources, project administration, supervision, writing – review and editing.

## Funding

The authors received no specific funding for this work.

## Ethics Statement

Ethical approval for this project was obtained from the Department of Research, Science, and Technology of the Ministry of Health and Sports of the Province of Mendoza (COPEIS; Resolution No. 149/2024).

## Consent

Written informed consent was obtained from all participants for participation in the co‐design workshops and for the use of photographs. Participants received detailed information about the research process before providing consent.

## Conflicts of Interest

The authors declare no conflicts of interest.

## Data Availability

Further data are available from the corresponding author upon reasonable request.
